# Combined Chemical Modification of Bamboo Material Prepared Using Vinyl Acetate and Methyl Methacrylate: Dimensional Stability, Chemical Structure, and Dynamic Mechanical Properties

**DOI:** 10.3390/polym11101651

**Published:** 2019-10-11

**Authors:** Saisai Huang, Qiufang Jiang, Bin Yu, Yujing Nie, Zhongqing Ma, Lingfei Ma

**Affiliations:** 1 School of Engineering, Zhejiang Provincial Collaborative Innovation Center for Bamboo Resources and High-Efficiency Utilization, Zhejiang A & F University, Hangzhou 311300, China; tanainai123456@gmail.com (S.H.); jiangqf8866@163.com (Q.J.); nieyujing@zafu.edu.cn (Y.N.); 2 Huzhou Ruiyi Wood Industry Co., Ltd., Huzhou 313220, China; yellowhuang332526@163.com

**Keywords:** bamboo, chemical modification, dimensional stability, dynamic thermodynamic, acetic anhydride, methyl methacrylate

## Abstract

Acetylation and in situ polymerization are two typical chemical modifications that are used to improve the dimensional stability of bamboo. In this work, the combination of chemical modification of vinyl acetate (VA) acetylation and methyl methacrylate (MMA) in situ polymerization of bamboo was employed. Performances of the treated bamboo were evaluated in terms of dimensional stability, wettability, thermal stability, chemical structure, and dynamic mechanical properties. Results show that the performances (dimensional stability, thermal stability, and wettability) of bamboo that was prepared via the combined pretreatment of VA and MMA (VA/MMA-B) were better than those of raw bamboo, VA single-treated bamboo (VA-B), and MMA single-treated bamboo (MMA-B). According to scanning electron microscopy (SEM) and Fourier transform infrared spectroscopy (FTIR) analyses, VA and MMA were mainly grafted onto the surface of the cell wall or in the bamboo cell lumen. The antiswelling efficiency and contact angle of VA/MMA-B increased to maximum values of 40.71% and 107.1°, respectively. From thermogravimetric analysis (TG/DTG curves), the highest onset decomposition temperature (277 °C) was observed in VA/MMA-B. From DMA analysis, the storage modulus (*E*’) of VA/MMA-B increased sharply from 15,057 Pa (untreated bamboo) to 17,909 Pa (single-treated bamboo), and the glass transition temperature was improved from 180 °C (raw bamboo) to 205 °C (single-treated bamboo).

## 1. Introduction

Bamboo is an important fast-growing and renewable material, and has been widely used as raw material to produce bamboo flooring, bamboo wood composite, bamboo building templates, and bamboo decorative materials because of its high mechanical properties and low strength-to-weight ratio [1,2]. However, bamboo use is highly limited by its strong hygroscopicity; specifically, the free hydroxyl groups from bamboo cell walls result in poor dimensional stability. Currently, many physical or chemical modification methods, such as heat treatment [3,4,5,6], in situ polymerization with organic monomers [7,8,9], acetylation treatment [10,11,12,13], and modification with 1,3-dimethylol-4,5- dihydroxyethyleneurea (DMDHEU) [14,15,16], have been used to improve its poor dimensional stability.

Acetylation is a conventional chemical modification method in which acetyl groups (CH_3_CO–) react with hydroxyl groups (–OH) linked to the cellulose of wood/bamboo material; therefore, it improves the dimensional stability of bamboo [12,13,17,18,19,20,21,22,23,24]. The traditional reagents used in wood/bamboo acetylation modification are acetic anhydride (AA), acetyl chlorides (AC), and thioacetic acid (TA) [12,13]. However, acetylation pretreated with these reagents produces a large amount of byproducts of strong acid, and this causes undesirable odors, strength loss, and corrosion of metal fasteners [25,26].

Recently, vinyl acetate (VA) was developed as an environmentally friendly reagent used in acetylation modification [23,27,28,29,30]. The byproduct produced from VA acetylation is acetaldehyde, which has a low boiling point and is easily removed. Several researchers have reported that the dimensional stability of wood treated via VA acetylation was much better than that of AA-treated wood. For example, Jebrane et al. reported that the weight percentage gain (WPG) of maritime pine after VA acetylation was 26.2%, which was higher than that of AA-treated wood (20.5%). Additionally, the swelling of blocks treated with VA, which have a WPG above 20%, was always less puffed than AA-treated wood [23]. Furthermore, it has been reported that the bonding strength between VA and cellulose is better than that between AA and cellulose, and this results in a lower swelling coefficient [29]. More VA than AA enters into the voids of cell walls, thereby decreasing the volume of voids and limiting the overall swelling of samples [28,30].

In situ polymerization of unsaturated polymer monomers (e.g., methyl methacrylate (MMA), styrene, acrylonitrile, and acrylamide) within wood pores (e.g., vessels, tracheids, capillaries, and ray cells) to fabricate wood polymer composites (WPCs) is another effective modification method for strengthening the mechanical properties of wood or for protecting the wood matrix from being attacked by water or microorganisms [31,32,33,34,35]. Methyl methacrylate (MMA) is one of the most important vinyl monomers used in WPCs because it has low viscosity and is relatively inexpensive [36,37,38,39,40]. Matto et al. reported that pinewood samples treated via in situ polymerization of MMA resulted in a higher retention of monomers and densification, less variation of permanent swelling, and higher mechanical resistance [37]. Fu et al. grafted MMA onto a wood surface using the atom transfer radical polymerization (ATRP) method, which resulted in the wood having better hydrophobicity [39]. Shang et al. found that the macromechanical properties (bending modulus and compressive modulus) of rattan were highly improved when MMA was grafted onto the surface of rattan [38].

As previously stated, studies reported in the literature have mainly focused individually on VA acetylation or MMA in situ polymerization of wood [23,27,32,37,39,41,42]. These two methods have been documented, and it has been concluded that both lead, to different extents, to improved dimensional stability and increased decay resistance of wood. However, the combined use of these two methods with respect to the enhancement of bamboo properties has not been reported. In this study, the effects of chemical modification via single use of VA and MMA on dimensional stability, chemical properties, and thermodynamic properties of bamboo were investigated. Then, a combined chemical modification method of VA acetylation and MMA in situ polymerization was employed to achieve a synergistic improvement in the dimensional stability and mechanical properties of bamboo.

## 2. Materials and Methods

### 2.1. Materials

Bamboo, purchased from Huzhou Ruiyi Wood Industry Co., Ltd. (Huzhou, China), was cut into samples with dimensions of 20 mm × 20 mm × 5 mm (L × W × H, respectively, for dimensional stability analysis) and 35 mm × 12 mm × 2.5 mm (L × W × H, respectively, for dynamic thermodynamic analysis). Samples were oven-dried at 105 °C for 12 h until constant weight was obtained. The sizes and weights of each specimen were then measured to calculate the volume (*V*_0_) and weight (*W*_0_).

### 2.2. Bamboo Acetylation Pretreatment

**Figure 1:**
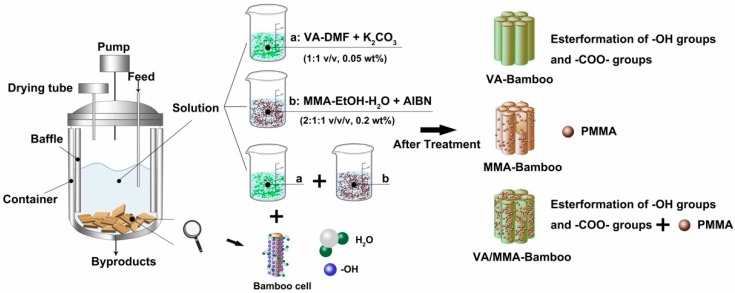
Schematic flow chart of bamboo acetylation, in situ polymerization of MMA on bamboo, and combined treatment of acetylation and in situ polymerization.

Vinyl acetate (VA) acetylation pretreatment of bamboo was carried out in a stainless steel container equipped with a vacuum pump, pressure pump, and calcium chloride drying tube (Figure 1). First, the bamboo specimen and vinyl acetate/dimethylformamide solution (1:1 V/V) with 0.5% concentration of potassium carbonate as the catalyst were added to the container. The container was then put under vacuum to −0.09 MPa, and this was maintained for 12 h. The container was then put into an oven and heated to 110 °C, which was maintained for 6 h. Finally, the acetylated samples were soaked in flowing water for 48 h to remove unreacted reagents and byproducts; then, the samples were dried in a vacuum oven under 0.01 MPa at 105 °C until a constant weight was obtained. The weight percentage gain (WPG) and the volume bulking efficiency (VBE) were calculated using Equations (1) and (2), respectively.

(1) $$WPG=\frac{W_{1}-W_{0}}{W_{0}}$$

(2) $$VBE=\frac{V_{1}-V_{0}}{V_{0}}$$

where *W*_1_ and *V*_1_ are respectively the absolute dry mass and volume of the samples before acetylation, and *W*_2_ and *V*_2_ are respectively the absolute dry mass and volume of the samples after acetylation.

### 2.3. In Situ Polymerization of MMA on Bamboo 

In situ polymerization of bamboo–MMA composite was carried out in the same container as mentioned above (Figure 1). First, MMA–EtOH solution (which was composed of MMA, ethanol, and deionized water with a volume ratio of 2:1:1) was added to the container. Azobisisobuttyronitrile (AIBN), which is an initiator with a relative molar ratio of 1:5, was then also added to the container. Next, under vacuum/pressure cycles for 12 h, VA-acetylated samples and untreated samples were impregnated with the MMA–EtOH solution in the container. All of the samples were then washed to remove the residual solvent. Finally, the samples were wrapped in aluminum foil and stored for 24 h; samples were placed separately into the oven at 80 °C for 6 h. In the end, the VA/MMA-treated bamboo and MMA-treated bamboo were vacuum-dried under 0.01 MPa at 105 °C to obtain constant weight. The conversion rate (CR) of MMA was calculated using Equation (3):

(3) $$CR=\frac{W_{3}-W_{0}}{W_{2}-W_{0}}$$

where *W*_3_ is the oven-dried weight after polymerization, *W*_2_ is the wet weight of bamboo after polymerization, and W_0_ is the absolute dry mass and volume of bamboo before all of the treatments.

### 2.4. Characterization of Raw and Pretreated Bamboo

The surface functional groups of raw and pretreated bamboo were tested using Fourier transform infrared spectroscopy (FTIR, Nicolet Is50, Thermo Fisher Scientific, Walthan, MA, USA). The surface morphologies of raw and pretreated bamboo were characterized using cold field emission scanning electron microscopy (SEM, SU8010, Hitachi, Chiyoda, Japan). The contact angles of raw and pretreated bamboo were measured using an interfacial tension tester (OCA200, DataPhysics, Filderstadt, Germany). The apparent contact angle was measured each second for the 5 s deposition on the surface of the sample. The thermal stabilities of raw and pretreated bamboo were tested using a thermogravimetric analyzer (TG-209, NETZSCH, Selb, Germany).

### 2.5. Dimensional Stability Analysis of Raw and Pretreated Bamboo

The dimensional stabilities of samples were evaluated using 12 specimens that were soaked in water for cyclic soaking–drying. Water soaking (at room temperature and under ambient pressure for 72 h) and oven-drying (103 °C for 24 h) were repeated three times. The weights and volumes of each sample were measured every cycle. Furthermore, the parameters of water absorption (*WA*), volume swelling efficiency (*S_w_*), volume shrinking efficiency (*S_k_*), and antiswelling efficiency (*ASE*) were calculated using Equations (4)–(7), respectively.

(4) $$WA=\frac{W_{wi}-W_{w0}}{W_{w0}}\times 100\%$$

(5) $$S_{w}=\frac{V_{wi}-V_{w0}}{V_{w0}}\times 100\%$$

(6) $$S_{k}=\frac{V_{w0}-V_{wi}}{V_{wi}}\times 100\%$$

(7) $$ASE=\frac{S_{wn}-S_{wn}^{’}}{S_{wn}^{’}}\times 100\%$$

where *W_wi_* and *V_wi_* are respectively the weight and size of a bamboo block after soaking it *i* times, and *W_w_*_0_ and *V_w_*_0_ are respectively the weight and size of a bamboo block after drying it *i* times. *i* is 1, 2, and 3. *S_wn_* is the swelling efficiency of untreated bamboo, and $S_{wn}^{\prime }$ is that of reacted bamboo.

### 2.6. Dynamic Mechanical Analysis of Raw and Pretreated Bamboo

The storage modulus (*E*’), loss modulus (*E*’’), and tan delta (tan δ) of both the raw and pretreated bamboo were recorded using a dynamic mechanical analyzer (DMA, Q800, TA Instruments, New Castle, DE, USA). The temperature was scanned from 40 to 240 °C, the heating rate was 2 °C/min, the frequency of the measurements was 1 Hz. Duplicate samples were measured to ensure the reproducibility of results.

## 3. Results

### 3.1. Weight Gain Rate, Volume Bulking Efficiency, and Conversion Rate

Table 1 shows the effects of using different modification methods on the weight gain rate (WPG) of bamboo, volume bulking efficiency (VBE) of bamboo, and conversion rate (CR) of MMA. WPG reached a maximum of 18.95% after the combined treatment of VA and MMA, and this indicates that more VA and MMA were fabricated on the bamboo. During the VA and MMA treatment process, VA has an active anhydride group that reacts with hydroxyl groups of bamboo components to create monoesters [28,29], and MMA in situ polymerizes on the surface or in a void of bamboo [23]. Ghorbani et al. reported that the WPG of poplar wood increased from 22.71% with the single treatment of maleic anhydride (MAN) and from 51.8% with the single treatment of MMA to 56.05% with the combined treatment of MAN and MMA [34].

Among the three chemical modification methods, the lowest value of VBE was obtained after MMA treatment because the majority of MMA entered the voids of cell walls rather than attached onto the surface of bamboo. In addition, the CR of MMA increased from 8.68% after MMA treatment to 20.69% after the combined treatment of VA and MMA, and this indicates that pretreating bamboo with VA was beneficial for increasing the CR of MMA. VA treatment enhanced interactions between the polymer and bamboo matrix, and more MMA filled the cell lumen of bamboo [41,43]. Li et al. reported that maleic anhydride successfully activated poplar through a nucleophilic substitution reaction and that this newly formed carboxyl group might act as a catalyst by providing a certain level of acidity for polymerization.

### 3.2. Morphology Characterization

**Figure 2:**
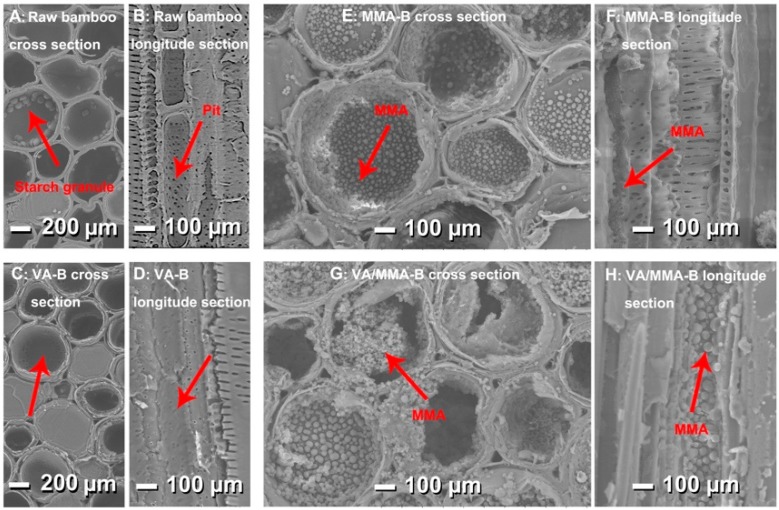
Morphology characterizations of the cross-section and longitudinal section of raw and pretreated bamboo: raw bamboo (**A**,**B**), VA-B (**C**,**D**), MMA-B (**E**,**F**), and VA/MMA-B (**G**,**H**).

Figure 2 shows morphology characterizations of the cross-section and longitudinal section of raw and pretreated bamboo. Compared to the raw bamboo (Figure 2A,B), the starch granules on the surface of the bamboo cells in the cross-section disappeared after VA treatment. Also, the pits on the parenchymal cells in the longitudinal section disappeared after VA treatment (Figure 2C,D), and this is because of the acetylic inflation [25]. Figure 2E,F clearly shows that MMA was in situ polymerized on the surface of the bamboo, and this is indicated by the tiny spherical granules. As seen in Figure 2G,H, more MMA was fabricated on the surface of the bamboo after the combined treatment of VA and MMA than was fabricated with just the VA treatment. In particular, abundant polymers filled most of the pores and pits in the cross-section and longitudinal section. It was expected that the use of VA would lead to penetration and swelling of the cell wall matrix and to a reduction in the number of hydroxyl groups. Pretreating the bamboo with VA could increase the adhesion amount of MMA on bamboo and create a cross-linked copolymer bonded onto the bamboo cell wall [32,34,37].

### 3.3. FTIR Analysis

**Figure 3:**
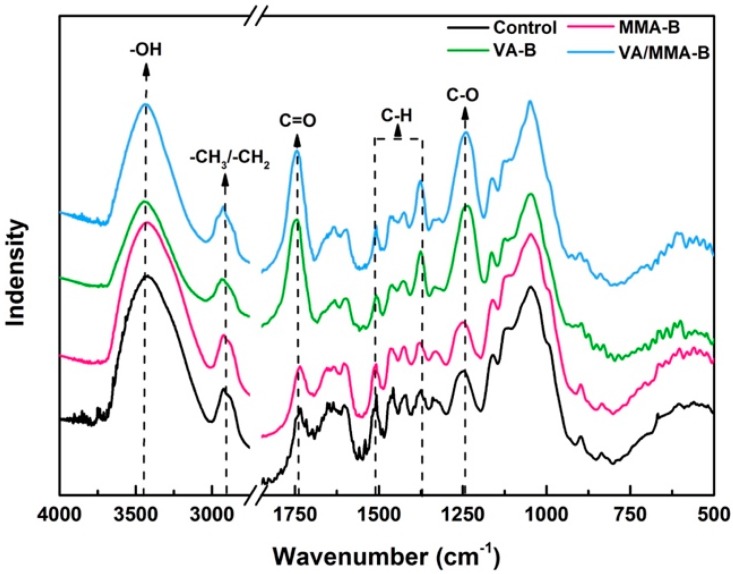
FTIR analysis of raw and pretreated bamboo.

Figure 3 shows the effects of chemical modification on the surface functional groups of bamboo. The band at 3405 cm^−1^ is attributed to the stretching vibration of the hydroxyl group [44]. Compared to the spectrum for raw bamboo, the intensity of the hydroxyl group remarkably decreased in the spectrum for the bamboo that was pretreated using VA and MMA. Hydrophilic hydroxyl groups in bamboo are esterified by acetyl groups (CH_3_CO–) in VA, and some of the hydroxyl groups are replaced in the polymer chains during the in situ polymerization process of MMA [37]. Among the three chemical modification methods, the lowest intensity band that corresponded to the hydroxyl group was observed in the spectrum for VA/MMA-B, and this indicates that the dimensional stability of VA/MMA-B was probably better than that of raw bamboo, VA-B, and MMA-B. The band at 2955 cm^−1^ is attributed to the stretching vibration of methyl (–CH_3_) and methylene (–CH_2_–) [45,46]. Obviously, the intensity of this band in the spectrum of VA/MMA-B is higher than that in the spectra of raw bamboo, MMA-B, and VA-B, and this indicates that more MMA was successfully grafted onto the bamboo cell walls after the combined treatment of VA and MMA [33,40]. The intensity of the band at 1745 cm^−1^ is ascribed to the stretching vibration of carbonyl groups (C=O) [47]. The intensity of the band for carbonyl groups in the spectrum of VA/MMA-B is much stronger than that in the spectra of raw bamboo and MMA-B, but it is slightly stronger than that in the spectrum of VA-B. This result indicates that quite a number of carbonyl groups from both VA and MMA molecules were grafted onto the bamboo cell walls of VA/MMA-B. Another slightly enhanced peak in the spectrum of VA/MMA-B is the band for the ester bond (C−O) stretching vibration at 1242 cm^−1^ [46]. This was caused by the successful reaction of bamboo hydroxyl groups with VA and MMA.

All of the treated bamboo samples were also analyzed using diffuse reflectance FTIR (DRIFT) spectroscopy to study the nonadsorbing matrix. Reflectance spectra were transformed to Kubelka–Munk (K-M) units to minimize scattering contributions to the absorption measured [48,49]. Changes in the relative intensities of bands in the spectra for raw or pretreated bamboo at 1740 cm^−1^, 1660 cm^−1^, 1506 cm^−1^, 1460 cm^−1^, 1422 cm^−1^, 1370 cm^−1^, 1240 cm^−1^, 1056 cm^−1^, and 899 cm^−1^ are given in Table 2. These variations in relative intensities of various bands are because of varying quantities of cellulose, hemicellulose, and lignin present in samples that were subjected to different treatments [33,50,51]. The band intensities for VA are always higher than those of the control sample. After MMA polymerization, the bands of VA/MMA-B for the aromatic skeletal vibrations at 1740 cm^−1^, 1660 cm^−1^, 1506 cm^−1^, and 1460 cm^−1^ increased in intensity. Likewise, the intensity of aromatic bending vibration at 1240 and 899 cm^−1^ increased. Regarding single MMA polymerization, the absorptions for C–H were similar with the raw sample and the band for C–H_2_ decreased. The results from DRIFT analysis indicated that acetylation bamboo increased the weight by VA acetylation and also achieved a sufficient chemical complex by MMA polymerization.

### 3.4. Dimensional Stability

**Figure 4:**
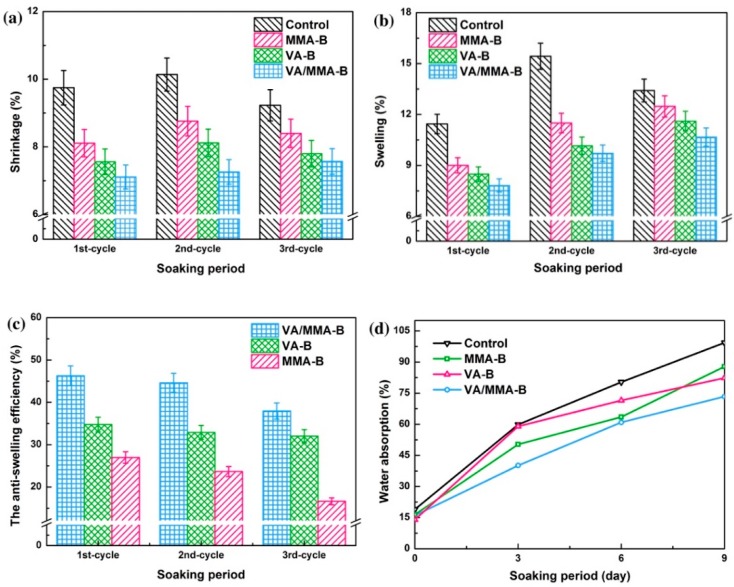
Dimensional stability of bamboo after different chemical modifications with three cycles of water soaking–drying experiments: volume shrinkage ratio (**a**), volume swelling ratio (**b**), antiswelling efficiency (**c**), and water absorption (**d**).

Figure 4 shows the effects of chemical modification on the dimensional stability of bamboo in the three cycles of water soaking–drying experiments. Figure 4 includes data for the volume swelling ratio, volume shrinkage ratio, antiswelling efficiency, and water absorption. Compared to control samples, the volume swelling ratio and volume shrinkage ratio of bamboo treated using the three chemical modification methods all decreased, and this indicates that the dimensional stability of bamboo treated using VA and MMA was improved. Among the three chemical modification methods, bamboo treated with the combination of VA and MMA exhibited the best dimensional stability. The antiswelling efficiency of VA/MMA-B reached a maximum value of 40.71%. We suggested that VA reacts with and deactivates the hygroscopic hydroxyl groups of the cell wall polymers and thereby creates a less polar particle surface, which is for better polymerization [30,34,40].

As reported in the literature, the hydrophilic hydroxyl groups are esterified by acetyl groups (CH_3_CO–) in VA, and this leads to a decreased availability of sites for hydrogen bonding. Furthermore, the voids of bamboo cell lumen are physically blocked via in situ polymerization of MMA, and this hinders the interaction between hydrophilic hydroxyl groups in bamboo and water in the environment [30,31,37]. With an increase in the number of times that the water soaking–drying experiment was repeated, the dimensional stability of bamboo that was treated using the three chemical modification methods decreased. This resulted in an increase in water absorption, as seen in Figure 4d. This observation is explained by the fact that part of the covering layer that formed between the bamboo surface and VA/MMA might be destroyed in the process of several rounds of the water soaking–drying experiment. A reason for this may be the partial loss of the polymer and a high physical cross-linking ratio for the treated samples during the water soaking–drying process. According to Li et al., the ASE of Poplar–PMGM–C also decreased after the third water immersion, and this is the same as our results [43]. Moreover, Zhang et al. indicated that the formed polymer also has a certain water absorption and hygroscopicity because of the high water absorption of the MMA monomer, and this may clarify our observation as well [36].

### 3.5. Wettability Analysis

**Figure 5:**
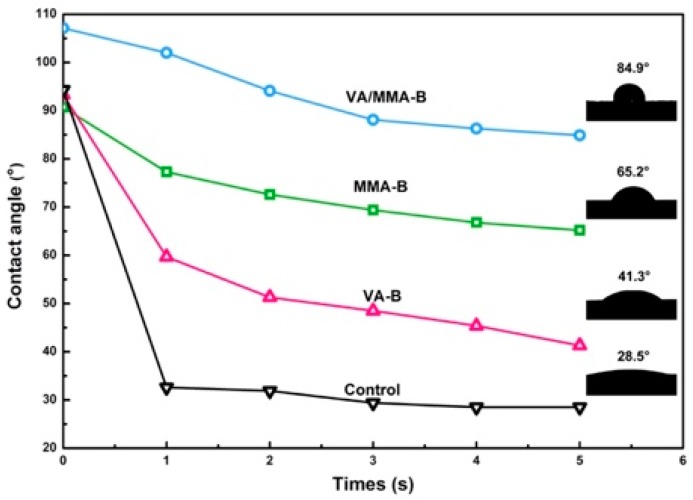
Contact angle profiles for the surfaces of raw and pretreated bamboo.

The wettability of raw and pretreated bamboo was evaluated using contact angle analysis (CA), where a large contact angle corresponds to greater hydrophobicity and better dimensional stability [52]. Figure 5 shows the transient profiles of the CA data of raw and pretreated bamboo. For the untreated samples, the initial CA was 94.3°, and then it sharply dropped to 28.5° with an increase in contact time from 1 s to 5 s. After chemical modification, the CA values of VA-B, MMA-B, and VA/MMA-B were 93.3–43.3°, 90.7–65.2°, and 107.1–84.9°, respectively, with varying contact times. Similar to CA data for raw bamboo, the CAs of treated bamboo gradually decreased with an increase in contact time. This result might have been caused by the surface defects of bamboo, where VA and MMA were not uniformly distributed. This defect makes it hard to obtain Young’s equilibrium CA, and the static CA might fluctuate within a range under real conditions [53].

Among the three chemical modification methods, VA/MMA-B had the highest CA value of 84.9–107.1°, indicating that VA/MMA-B had the greatest hydrophobicity and best dimensional stability. The hydrophilic functional groups were greatly reduced by VA acetylation and in situ polymerization of MMA [23,43]. This result indicates that the combined treatment of VA and MMA is an efficient method for improving the dimensional stability and polymer coverage of the bamboo surface [39].

### 3.6. TG Analysis

**Figure 6:**
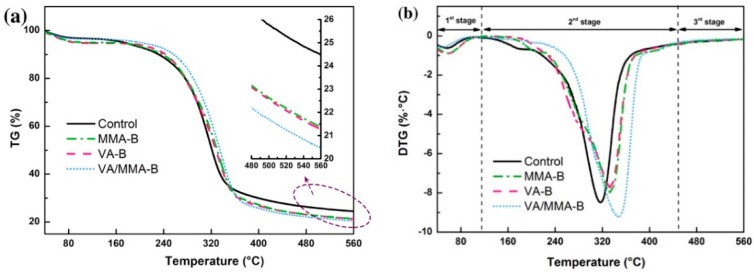
Thermal degradation behaviors of raw and pretreated bamboo: TG (**a**) and DTG (**b**) curves.

Figure 6 shows the thermal degradation behaviors of raw and pretreated bamboo under a nitrogen atmosphere at a heating rate of 10 °C/min. According to the TG curves, the highest residual mass was observed in raw bamboo (24.5%), followed by MMA-B (21.4%), VA-B (21.3%), and VA/MMA-B (20.4%). The thermal stability of VA and MMA was lower than that of raw bamboo. Therefore, with an increase in the WPG of treated bamboo, the residual mass decreased, but the weight loss rate of the treated bamboo increased.

According to the DTG curves, the thermal degradation process of bamboo can be divided into three stages: the moisture evaporation stage (50–150 °C), fast devolatilization stage (150–450 °C), and carbonization stage (450–650 °C) [44,46,54,55,56]. In the fast devolatilization stage, the peak temperatures of the maximum weight loss for raw bamboo, MMA-B, VA-B, and VA/MMA-B were 317 °C, 334 °C, 333 °C, and 337 °C, respectively. This gradually moved toward the side of higher pyrolysis temperature with an increase in the extent of pretreatment via the chemical modifications. This result indicates that VA/MMA-B had enhanced thermal stability compared to all of the other samples. The esterification reaction between VA and the hydroxy groups in bamboo resulted in an increase in the degree of the crystallinity of cellulose, and this was in good agreement with the results from Wei et al. [51]. The decrease in residual mass after grafting MMA was because of the presence of MMA, which degraded more easily than bamboo [42].

### 3.7. Dynamic Mechanical Analysis

**Figure 7:**
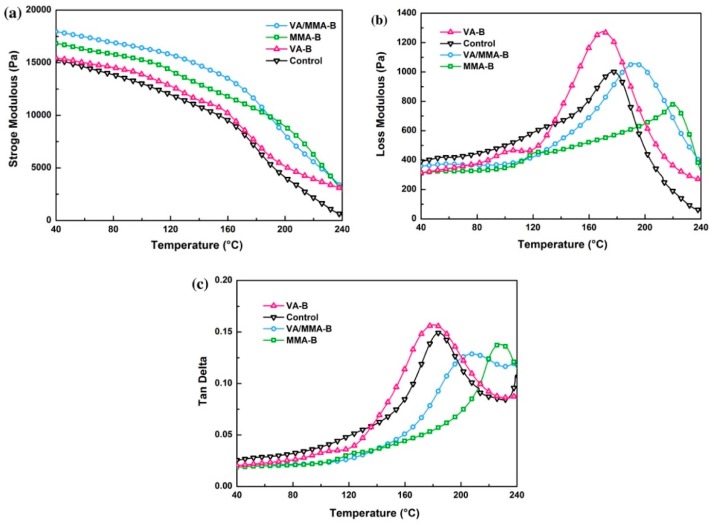
Storage modulus (*E*’) (**a**), loss modulus (*E*’’) (**b**), and loss tangent (tan *δ*) (**c**) curves of raw and treated bamboo.

Variations in the dynamic storage modulus (*E*’), loss modulus (*E*’’), and loss tangent (tan δ) of raw and pretreated bamboo are shown in Figure 7. The dynamic storage modulus is widely used to assess the load-bearing capability of a composite material [57]. The storage modulus of raw bamboo was about 15,057 Pa. In general, the storage modulus (*E*’) of all of the bamboo samples decreased with an increase in temperature. It is worth noting that the value of *E*’ for VA/MMA-B was the highest at the same temperature for each of the four samples (17,909 Pa), and this indicates that the dynamic storage modulus was enhanced after the combined pretreatment using VA and MMA. With an increase in the concentration of acetyl and methyl groups, intermolecular hydrogen bonding was broken. A certain number of hydroxyl groups were then regenerated, and the cellulose chains were consequently closer [32,33,41]. It is believed that the stiffness of the bamboo fibers was enhanced, and the initial storage modulus value of the treated samples is also reflected by this. A similar result has been reported by Jebrane et al. Some esterified material expands into the micropores or lumen of the bamboo cell wall after treatment, and this results in a higher value of *E*’ [23].

Figure 7b illustrates the temperature spectrum of the loss modulus (*E*’’) for raw and pretreated bamboo over the entire temperature range. The highest value of *E*’’ was obtained for VA-B (1275.4 Pa at 170 °C), and the lowest value was for MMA-B (785.5 Pa at 220 °C). With an increase in the in situ polymerization of MMA on bamboo, intermolecular friction and energy consumption of bamboo also increased. Furthermore, a decrease in *E*’’ is consistent with some internal plasticization occurring after polymerization, and this causes a decrease in the energy required to initiate chain mobility [58]. Researchers have reported that the thermal-softening temperature of lignin, hemicellulose, and cellulose are 30–205 °C, 150–220 °C, and 200–250 °C, respectively [59,60]. The loss peak at a temperature of 200 °C is labeled as an α relaxation process that is derived from micro-Brownian motions of bamboo cell wall polymers in the noncrystalline region [28,59]. After chemical modification, there was a remarkable difference in the α relaxation process for different samples. Compared to raw bamboo, the in situ polymerization of MMA on bamboo (MMA-B and VA/MMA-B) led to higher temperature of the α-peak because more MMA, which has amorphous chains, was grafted onto the bamboo. However, acetylation with VA did not significantly influence the temperature of the α-peak.

Figure 7c shows the glass transition temperature for raw and pretreated bamboo in terms of the mechanical loss factor (tan δ). The glass transition temperature (*T_g_*) was approximately equal to the temperature when tan *δ* reached its maximum value. After in situ polymerization of MMA on bamboo, the glass transition temperature increased from 180 °C for raw bamboo to 205 °C for VA/MMA-B and 220 °C for MMA-B. The increased glass transition temperature should be ascribed to the reinforcement of polymer on bamboo as caused by the in situ polymerization of MMA in cell lumen [33]. However, compared to the glass transition temperature for raw bamboo, a slight decrease was observed in the glass transition temperature for VA-B (175 °C).

## 4. Conclusions

The effects of the combined treatment with VA and MMA on dimensional stability, chemical structure, and dynamic mechanical properties of bamboo were systematically investigated. Results show that the dimensional stability (i.e., antiswelling efficiency and water absorption) of bamboo after the combined treatment of VA and MMA was remarkably improved because of the decrease in hydrophilic hydroxyl groups. VA and MMA were mainly grafted onto the surface of the cell walls or in the bamboo cell lumen. From TG analysis, an increase in the extent of pretreatment via chemical modifications resulted in the peak temperatures of the maximum weight loss gradually moving toward the side of higher pyrolysis temperature. From DMA analysis, compared to untreated and single-treated bamboo, the storage modulus (*E*’) of VA/MMA-B sharply increased by about 3 MPa, and the glass transition temperature increased from 180 °C to 205 °C. At the same time, the glass transition temperature of MMA-B was the highest (220 °C).

## Figures and Tables

**Table 1:** Weight percentage gain, volume bulking efficiency, and conversion rate of treated bamboo.

Samples	Weight Percentage Gain (%)	Volume Bulking Efficiency (%)	Conversion Rate (%)
VA-B	11.09 ± 0.56	4.25 ± 0.21	/
MMA-B	6.59 ± 0.33	3.49 ± 0.17	8.68 ± 0.43
VA/MMA-B	18.95 ± 0.95	8.66 ± 0.43	20.69 ± 1.03

**Table 2:** DRIFT spectra analysis of raw and pretreated bamboo.

Wavenumber/(cm^−1^)	Assignment	Peak Height of Associated Bands			
		Control	VA/MMA-B	VA-B	MMA-B
1740	C=O stretching vibration	0.195	0.766	0.494	0.323
1660	H–O–H deformation vibration and conjugated C=O stretching vibration	0.159	0.486	0.440	0.164
1506	Aromatic skeletal	0.171	0.446	0.377	0.171
1460	C–H deformation (asymmetric) and benzene vibration in lignin	0.190	0.421	0.457	0.176
1422	C–H deformation (asymmetric)	0.189	0.457	0.457	0.189
1370	C–H_2_ deformation (symmetric)	0.454	0.530	0.229	0.187
1240	C–O stretching vibration in lignin, acetyl and carboxylic vibration in xylan	0.180	0.587	0.470	0.248
1056	C–O stretching	0.177	0.526	0.472	0.231
899	C1 group frequency in cellulose and hemicellulose	0.077	0.216	0.132	0.065
